# Bone Morphogenetic Protein 6 Inhibits the Immunomodulatory Property of BMMSCs via Id1 in Sjögren's Syndrome

**DOI:** 10.1155/2018/9837035

**Published:** 2018-08-02

**Authors:** Yingying Su, Yi Gu, Ruiqing Wu, Hao Wang

**Affiliations:** ^1^Department of Stomatology, Beijing Tiantan Hospital, Capital Medical University, Beijing, China; ^2^Department of Pediatrics, Beijing Chaoyang Hospital, Capital Medical University, Beijing, China

## Abstract

Mesenchymal stem cells (MSCs) treatment has emerged as a promising approach for treating Sjögren's syndrome (SS). Impaired immunoregulatory activities of bone marrow mesenchymal stem cells (BMMSCs) are found in both SS patients and animal models, and the underlying mechanism is poorly understood. Increased expression of BMP6 is reported to be related to SS. The aim herein was to determine the effects of BMP6 on BMMSCs function. BMMSCs were isolated from SS patients and NOD mice and showed a high level of BMP6 expression. The effects of BMP6 on BMMSCs function were investigated using *in vitro* BMMSCs differentiation and *in vitro* and *in vivo* T cell proliferation and polarization assays. BMP6 increased osteogenic differentiation of BMMSCs and inhibited the immunomodulatory properties of BMMSCs. BMP6 enhanced T cell proliferation and Th1/Th17 differentiation in a T cell-BMMSC coculture system. Mechanistically, BMP6 downregulated PGE2 and upregulated IFN-gamma via Id1 (inhibitor of DNA-binding protein 1). Neutralizing BMP6 and knockdown of Id1 could restore the BMMSCs immunosuppressive function both *in vitro* and *in vivo*. The present results suggest a novel role of Id1 in BMP-mediated MSCs function, which may contribute to a better understanding of the mechanism of action of MSCs in treating autoimmune diseases.

## 1. Introduction

Sjögren's syndrome (SS) is a chronic and systemic autoimmune disease that mainly affects middle-aged women. SS is characterized by lymphocytic infiltration of salivary and lacrimal glands, leading to dry eyes and mouth [1, 2]. The etiology of SS is not fully delineated, and some genetic and environmental factors are suggested to be involved [1]. Current treatment strategies include punctual occlusion, artificial tears, use of saliva substitutes, and pharmacological therapy such as anti-inflammatory agents and immunosuppressive drugs [3]. However, these strategies are considered unable to modify the course of the disease and remain symptom-based [4].

In recent years, an accumulating body of evidence has supported the promising effects of mesenchymal stem cells (MSCs) in the treatment of autoimmune diseases. MSCs are multipotent stem cells characterized by colony-forming ability, multilineage differentiation, and self-renewal capacity [5]. Importantly, MSCs have an immunosuppressive capacity that enables their role in the treatment of a variety of immune diseases. Impaired immunoregulatory activities of MSCs are found in both SS patients and animal models [6]. MSCs treatment can suppress autoimmunity and restore salivary and lacrimal gland secretory function in both animal models and SS patients [6, 7]. However, the underlying mechanisms responsible for the impaired immunoregulatory function of MSCs in SS remain unclear. Elevated expression of bone morphogenetic protein 6 (BMP6) was recently reported in the epithelia of SS patients, and hypofunction and increased lymphocytic infiltration of the salivary gland were induced by the overexpression of BMP6 in normal mice [8]. Whether BMP6 is involved in the dysfunction of MSCs immunoregulatory capacity is unclear. In the present study, we found that BMP6 impaired immunomodulatory properties of normal BMMSCs via Id1 and anti-BMP6 treatment, and blockage of Id1 rescued BMMSC function.

## 2. Materials and Methods

### 2.1. Animals

Female NOD/Ltj (referred to as NOD) mice were purchased from Beijing HFK Bioscience Co. and served as the SS animal model. OT-II transgenic mice, CD45.1 transgenic mice, and BALB/c mice were obtained from the Institute of Laboratory Animal Science, Chinese Academy of Medical Sciences. Animals were housed in a specific pathogen-free animal facility. All the animal procedures were approved by the Animal Care and Use Committee of Capital Medical University.

### 2.2. BMMSC Isolation and Culture

Total mRNA of BMMSCs from six SS patients and nine healthy volunteers was obtained from Drum Tower Hospital of Nanjing University Medical School. Mouse BMMSCs were isolated and cultured as described previously [9]. In brief, bone marrow cells were flashed out from bone cavity of femurs and tibias with 2% heat-inactivated fetal bovine serum (FBS; Gibco, USA) in phosphate-buffered saline (PBS; Hyclone, China). Single-cell suspension was obtained by passing bone marrow cells through a 70 mm cell strainer (BD Bioscience, USA), and cells were then seeded onto 100 mm culture dishes (Corning, USA). After culture for 14 days, colony-forming attached cells were passaged for further experiments. To confirm the BMMSC phenotypes, flow cytometric analysis was used to ensure that the cells were positive for CD90, CD105, and CD146 and negative for CD34 and CD45.

### 2.3. BMMSC *In Vitro* Differentiation Assays

To assess the osteogenic ability of BMMSCs, BMMSCs were cultured in osteoinductive medium containing 10 mM *β*-glycerophosphate (Sigma, USA), 100 *μ*M L-ascorbic acid 2-phosphate (Sigma), and 10 nM dexamethasone (Sigma, USA). After 7 days of induction, the cells were stained with an alkaline phosphatase (ALP) staining kit (Sigma, USA) or collected for ALP activity testing using alkaline phosphatase yellow (pNPP) liquid ELISA substrate (Sigma, USA). For the adipo-induction, an adipogenic differentiation kit (Invitrogen, USA) was used. After 4 weeks of induction, the cells were stained with Oil Red O (Sigma, USA). After photographing, lipid droplets were then dissolved with 100% isopropanol, and OD value was measured at 492 nm. For real-time RT-PCR assays, total mRNA was isolated from BMMSCs after one week of induction. All assays were done in duplicate from at least three independent experiments.

### 2.4. Coculture of BMMSCs and T Cells and T Cell Proliferation Assay

Mouse lymph nodes were derived from BALB/c mice. T cells were isolated from lymphocytes using a naïve CD4^+^ T Cell isolation kit (Miltenyi Biotec, German) following the manufacturer's instructions. The proliferation of CD4^+^ T cells was detected using carboxyfluorescein succinimidyl ester (CFSE; Invitrogen, USA) labeling according to the manufacturer's instructions. To activate naïve CD4^+^ T cells, naïve CD4^+^ T cells were stimulated with immobilized 5 *μ*g/ml anti-mouse CD3 antibody and 1 *μ*g/ml anti-mouse CD28 (BD Pharmingen, USA) for 2-3 days. Activated CD4^+^ T cells were then cocultured with BMMSCs from NOD or BALB/c mice at the ratio of 1 : 1 for 3 days. The proliferation of T cells was determined by loss of CFSE fluorescence.

### 2.5. Real-Time RT-PCR

Total RNA was isolated from BMMSCs using Trizol reagents (Invitrogen, USA), and cDNA was synthesized using the PrimeScript™ RT reagent kit (Takara, Dalian, China). Real-time RT-PCR was analyzed using SYBR *Premix Ex Taq™* (Takara, Dalian, China). The following primers were used: *BMP6* (Hs01099594_m1, Invitrogen), *GAPDH* (Hs02786624_g1, Invitrogen), *Bmp6* (Mm01332882_m1, Invitrogen), *Gapdh* (Mm99999915_g1, Invitrogen), *Bglap* (Mm03413826_mH, Invitrogen), *Alp* (Mm01187117_m1, Invitrogen), *Pparg* (Mm00440940_m1, Invitrogen), *Fabp4* (Mm00445878_m1, Invitrogen), *Ido1* (Mm00492590_m1, Invitrogen), *Nos2* (Mm00440502_m1, Invitrogen), *TGFb1* (Mm00441727_g1, Invitrogen), *Id1* (Mm00775963_g1, Invitrogen), *Id2* (Mm00711781_m1, Invitrogen), *Id3* (Mm00492575_m1, Invitrogen), *Id4* (Mm00499701_m1, Invitrogen), and *Cox2* (Mm03294838_g1, Invitrogen).

### 2.6. Enzyme-Linked Immunosorbent Assay (ELISA)

Mice peripheral blood serum was collected from the retro-orbital plexus. The salivary gland was grounded with protein extraction reagent; the ground tissue was then centrifuged, and the supernatant was collected for cytokine measurement. The cytokine levels of IFN-gamma, IL-17, and PGE2 were measured by using a mouse ELISA kit (R&D Systems, USA) according to the manufacturer's instructions.

### 2.7. BMP6 Neutralization Antibody Treatment

BMP6 present in the T cell and BMMSCs coculture system was neutralized using an anti-mouse BMP6 antibody (R&D Systems, USA) at a concentration of 10 *μ*g/ml/5 × 10^5^ cells. Monoclonal rat IgG2B (R&D Systems, USA) served as a control.

### 2.8. siRNA Transfection

Id1 siRNA (sc-35632, Santa Cruz, USA) and the control scrambled sequence siRNA (sc-36869, Santa Cruz, USA) were transfected using Lipofectamine™ 2000 (Invitrogen, USA). Twenty-four or 48 hours after transfection, the cells or the supernatants were collected for further experiments.

### 2.9. *In Vivo* Immunosuppression

Naïve CD4^+^ T cells were isolated and purified from the spleen and lymph nodes of OT-II transgenic mice. Approximately 1 × 10^6^ CFSE-labeled naïve CD4^+^ T cells and 5 × 10^5^ BMMSCs were cotransferred, by tail vein injection, to recipient CD45.1 transgenic C57BL/6 mice. Four hours later, 25 *μ*g OVA (Sigma-Aldrich, USA) emulsified in the complete Freund's adjuvant (CFA) was injected into the footpad of recipient mice. The popliteal lymph nodes of recipient mice were removed, and flow cytometry was carried out to measure CD45.2^+^ T cell proliferation and differentiation.

### 2.10. Flow Cytometry

Cell suspensions from the spleen and lymph nodes were stained with fluorescence-conjugated anti-CD4, anti-IFN-gamma, and anti-IL-17 (BD Biosciences, USA) for analysis of CD4^+^ T, Th1, and Th17 cells.

### 2.11. Statistical Analyses

The salivary flow rates were analyzed with repeated measurement, and other data were analyzed by Student's *t*-test or one-way analysis of variance analysis. All data are presented as the mean ± SEM (infiltrating area statistics) or SD (other data). Analysis was performed using SPSS 13.0 Software. A *P* value less than 0.05 was considered statistically significant.

## 3. Results

### 3.1. BMP6 Was Overexpressed in BMMSCs of SS Patients and NOD Mice and Regulated BMMSCs Differentiation

**Figure 1:**
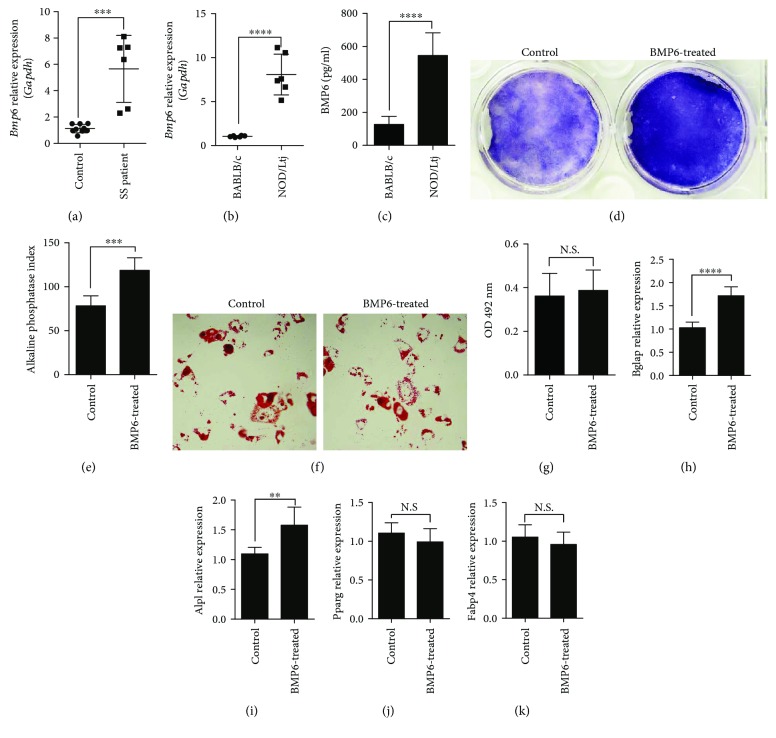
BMP6 was overexpressed in BMMSCs of SS patients and NOD mice and regulated BMMSCs differentiation. Real-time RT-PCR results indicated that the mRNA level of BMP6 is about five times higher in SS patient BMMSCs (a) and eight times higher in NOD BMMSCs (b) than in normal BMMSCs (^∗∗∗^ *P* < 0.001, ^∗∗∗∗^ *P* < 0.0001). (c) ELISA showed that the BMP6 protein level is higher in the supernatant of NOD BMMSCs culture medium compared with BALB/c (^∗∗∗∗^ *P* < 0.0001). (d, e) BMP6-treated BMMSCs showed a significant increase in ALP activity compared to untreated controls (^∗∗∗^ *P* < 0.001). Real-time RT-PCR analysis demonstrated elevated expression of BGLAP mRNA (h) and ALP mRNA (i) in BMP6-treated cells (^∗∗^ *P* < 0.01, ^∗∗∗∗^ *P* < 0.0001). (f, g) Oil Red O staining showed that BMP6 has no effect on cellular lipid accumulation in BMMSCs. Real-time RT-PCR results revealed that BMP6 do not increase the expression of PPAR*γ* mRNA (j) and FABP4 mRNA (k). N.S.: no significant difference.

BMP6 was reported to be overexpressed in the epithelia of salivary glands of SS patients and NOD mice [8]. Here, we first investigated BMP6 expression in BMMSCs of SS patients and NOD mice. Real-time RT-PCR results indicated that the mRNA level of *Bmp6* is about five times higher in SS patient BMMSCs and eight times higher in NOD BMMSCs than in normal BMMSCs (Figures 1(a) and 1(b)). The elevated BMP6 protein in the supernatant of NOD BMMSCs culture medium when compared with BALB/c was also detected by ELISA (Figure 1(c)). We next treated BALB/c BMMSCs with BMP6 to examine its role in the differentiation of BMMSCs. BMMSCs cultured with BMP6 showed a significant increase in ALP activity relative to untreated controls (Figures 1(d) and 1(e)). This effect was confirmed by real-time RT-PCR analysis. Expression of osteogenic markers ALP and BGLAP (bone gamma-carboxyglutamate protein) showed a significant increase in BMP6-treated cells (Figures 1(h) and 1(i)). After adipogenic induction, BMMSCs treated with BMP6 showed no detectable difference in cellular lipid accumulation from control cells, as evidenced by Oil Red O staining (Figures 1(f) and 1(g)). Consistently, expression of adipogenesis-induced genes, including PPAR*γ* (peroxisome proliferator-activated receptor-*γ*) and FABP4 (fatty acid-binding protein 4) demonstrated no significant changes in the presence of BMP6 (Figures 1(j) and 1(k)).

### 3.2. BMP6 Impaired Immunomodulatory Properties of BMMSCs by Downregulating PGE2 and Upregulating Th1 and Th17 Cells

**Figure 2:**
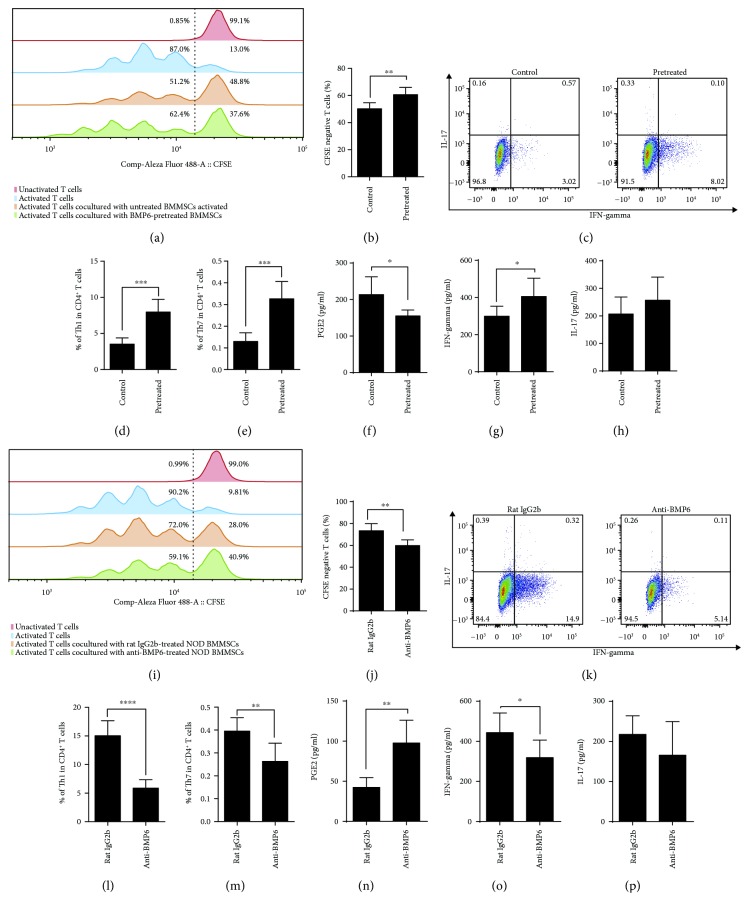
BMP6 impaired the immunomodulatory properties of BMMSCs by downregulating PGE2 and upregulating Th1 and Th17 cells. (a, b) CFSE assays showed T cells proliferation was inhibited by BMMSCs, and this effect could be attenuated by BMP6 (^∗∗^ *P* < 0.01). (c, d, e) Flow cytometric analysis indicated that BMP6 significantly inhibited BMMSC-mediated downregulation of Th1 and Th17 cells (^∗∗∗^ *P* < 0.001). ELISA assays revealed that BMP6-treated BMMSCs showed a decreased concentration of PGE2 (f) and increased concentration of IFN-gamma (g) in the supernatants of the BMMSCs/T cell coculture system (^∗^ *P* < 0.05). (h) Although no significant difference was detected, there was an increasing trend in IL-17 concentration in the supernatants of the BMMSCs/T cell coculture system (i, j). CFSE assays showed that T cells proliferated less when cocultured with anti-BMP6-pretreated NOD BMMSCs compared with the isotype antibody group (^∗∗^ *P* < 0.01). (k, l, m) Flow cytometric analysis showed that anti-BMP6-treated NOD BMMSCs exert an effect on downregulating Th1 and Th17 cells (^∗∗^ *P* < 0.01, ^∗∗∗∗^ *P* < 0.0001). ELISA assays revealed that anti-BMP6-pretreated NOD BMMSCs significantly increased the levels of PGE2 (n) and decreased the levels of IFN-gamma (o) but had no effect on IL-17 (p) in the supernatants of the BMMSCs/T cell coculture system (^∗^ *P* < 0.05, ^∗∗^ *P* < 0.01).

To investigate whether BMP6 treatment impaired the immunomodulatory properties of BMMSCs, activated T cells were cocultured with BMMSCs for 48 hours. Proliferation of T cells was inhibited by normal BMMSCs from 87% to 51.2%, while BMP6-treated BMMSCs could only inhibit T cell proliferation by 62.4% (Figures 2(a) and 2(b)). Normal BMMSCs can regulate T cell differentiation into Th1 and Th17 cells, but BMP6 treatment significantly inhibited BMMSC-mediated downregulation of Th1 and Th17 cells (Figures 2(c)–2(e)). ELISA revealed that BMP6-treated BMMSCs showed a decreased concentration of PGE2 (Figure 2(f)) and increased concentration of IFN-gamma (Figure 2(g)) in the supernatants of the BMMSCs/T cell coculture system. Although no significant difference was detected, there was an increasing trend in IL-17 concentration in the supernatants of the BMMSCs/T cell coculture system (Figure 2(h)). In contrast, BMP6-neutralizing antibodies (anti-BMP6) could significantly enhance the immunomodulatory properties of BMMSCs from NOD mice. T cells proliferated less when cocultured with anti-BMP6-pretreated NOD BMMSCs compared with the isotype antibody group (Figures 2(i) and 2(j)). Furthermore, anti-BMP6-pretreated NOD BMMSCs showed an increased effect on downregulating Th1 and Th17 cells (Figures 2(k)–2(m)). Consistently, anti-BMP6-pretreated NOD BMMSCs significantly increased the levels of T cell-produced PGE2 (Figure 2(n)) and decreased the levels of IFN-gamma (Figure 2(o)), but had no effect on IL-17 (Figure 2(p)) in the supernatants of the BMMSCs/T cell coculture system.

### 3.3. BMP6 Impaired Immunomodulatory Properties of BMMSCs and Downregulated PGE2 via Id1

**Figure 3:**
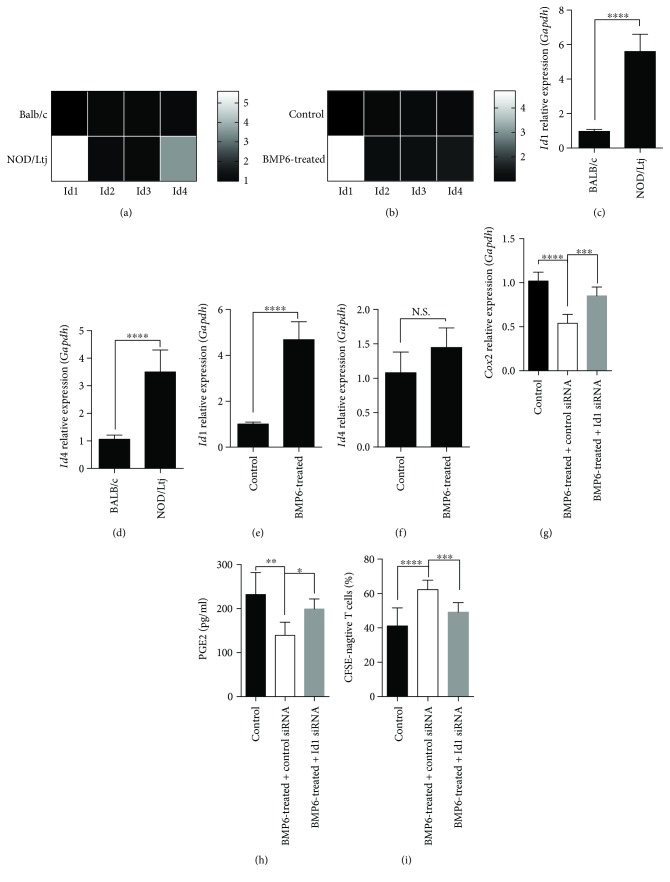
BMP6 impaired immunomodulatory properties of BMMSCs and downregulated PGE2 via Id1. (a, c, d) Real-time RT-PCR results demonstrated a higher level of Id1 and Id4 mRNA expression in BMMSCs derived from NOD mice compared with BALB/c, while Id2 and Id3 transcripts showed no significant difference between two groups (a) (^∗∗∗∗^ *P* < 0.0001). (b, e, f) BMP6-treated BALB/c BMMSCs showed an increase in Id1, rather than in Id2, Id3, or Id4 mRNA expression (^∗∗∗∗^ *P* < 0.0001) Knockdown of Id1 increased BMP6-induced Cox2 (g) and PGE2 (h) downregulation, as evidenced by real-time RT-PCR and ELISA, respectively. (^∗^ *P* < 0.05, ^∗∗^ *P* < 0.01, ^∗∗∗^ *P* < 0.001, ^∗∗∗∗^ *P* < 0.0001). (i) CFSE assays indicated that BMP6 inhibited downregulation of T cells proliferation mediated by BMMSCs, while Id1 knockdown attenuated this effect (^∗∗∗^ *P* < 0.001, ^∗∗∗∗^ *P* < 0.0001). N.S.: no significant difference.

Given that Ids are suggested to be the main targets of BMPs [10], we predicted that Ids were involved in BMP6-mediated impairment of BMMSCs immunoregulatory function. Real-time RT-PCR results demonstrated a higher level of *Id1* and *Id4* mRNA expression in BMMSCs derived from NOD mice compared with BALB/c mice (Figures 3(a), 3(c), and 3(d)), while *Id2* and *Id3* transcripts showed no significant difference between the two groups (Figure 3(a)). When treated with BMP6, BALB/c BMMSCs showed an increase in *Id1*, rather than in *Id2*, *Id3*, or *Id4* mRNA expression (Figures 3(b), 3(e), and 3(f)). To evaluate the effects of Id1 on BMMSC immunoregulatory function at the molecular level, we examined the expression levels of PGE2 and its synthase Cox2 in BMMSCs treated with or without BMP6 or Id1 siRNA. Knockdown of Id1 increased BMP6-induced Cox2 (Figure 3(g)) and PGE2 (Figure 3(h)) downregulation. In addition, Id1 knockdown dramatically increased the inhibitory capacity of BMMSCs on T cells proliferation reduced by BMP6 (Figure 3(i)). Our results suggested that Id1 mediated BMP6-induced impairment of BMMSC immunomodulatory properties by downregulating PGE2.

### 3.4. Knockdown of Id1 Rescued Impaired Immunosuppressive Capacity of BMMSCs Induced by BMP6 *In Vivo*

**Figure 4:**
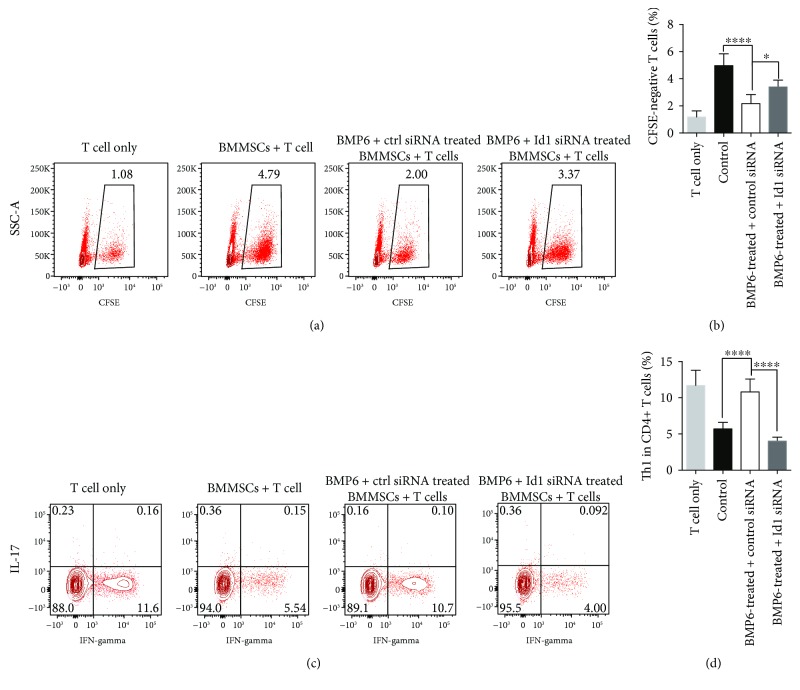
Knockdown of Id1 rescued impaired immunosuppressive capacity of BMMSCs induced by BMP6 *in vivo*. (a, b) BMMSCs treatment significantly reduced the number of CD4^+^ T cells, while BMP6-treated BMMSCs showed no significant reduction. When Id1 was blocked by siRNA in BMP6-treated BMMSCs, a greater reduction in CD4^+^ T cells number was observed (^∗^ *P* < 0.05, ^∗∗∗∗^ *P* < 0.0001). (c, d) A greater ratio of Th1 cells were detected in the presence of BMP6 compared with BMMSCs alone. Knockdown of Id1 significantly decreased the proportion of Th1 cells. (^∗∗∗∗^ *P* < 0.0001).

To further confirm the role of Id1 in BMP6-induced BMMSC immunosuppressive function impairment, we adoptively transferred OT-II T cells plus BMMSCs treated with or without BMP6 or Id1 siRNA to CD45.1 transgenic C57BL/6 mice. BMMSC treatment significantly reduced the number of proliferated CD4^+^ T cells, while BMP6-treated BMMSCs showed no significant reduction. When Id1 was blocked by siRNA in BMP6-treated BMMSCs, a greater reduction in CD4^+^ T cell number was observed (Figures 4(a) and 4(b)). The effects of Id1 on BMMSC-induced differentiation of T cells *in vivo* were also checked. A greater ratio of Th1 cells was detected in the presence of BMP6 compared with BMMSCs alone. Knockdown of Id1 significantly decreased the proportion of Th1 cells (Figures 4(c) and 4(d)). Together, these data showed that knockdown of Id1 could rescue impaired immunosuppressive capacity of BMMSCs induced by BMP6 *in vivo*.

## 4. Discussion

MSCs have been known to have immunoregulatory functions for decades [11–16], and it was reported that MSCs from many autoimmune disease patients, including those with SS, have deficient immunoregulatory functions and biological properties [6, 17, 18], which was one of the critical mechanisms for “MSCs therapy”. In the present study, we found that the differentiation potentials and immunoregulatory activities of BMMSCs are impaired in SS patients and disease mice. BMP6 was expressed at a higher level in BMMSCs derived from SS patients as well as NOD mice and regulated BMMSCs function, especially the immunomodulatory properties via Id1. Neutralizing the BMP6 and knockdown Id1 significantly restored the BMMSCs function both *in vitro* and *in vivo*.

BMP6 was reported to be highly expressed in the epithelia of the salivary gland in patients with SS, and overexpression of BMP6 locally could induce loss of cellular water permeability and salivary gland hypofunction [2, 8]. However, proinflammatory cytokines or autoantibodies associated with SS after BMP6 overexpression locally were not found, which suggests that the loss of salivary gland activity in SS patients may result from changes in epithelial function by BMP6 expression rather than the direct immune response [8]. In the present study, for the first time, we found that BMP6 was also highly expressed in BMMSCs from SS patients and animal models. BMP6 treatment can improve the osteogenic differentiation but impair the immunoregulatory function of BMMSCs. These results indicate that BMP6 could not lead to the dysfunction of the salivary gland but may influence the tissue repair and immune functions of systemic MSCs, which may collectively contribute to the inflammation of the salivary gland in SS.

BMMSCs from NOD mice were reported to have lower osteogenic and adipogenic differentiation capacity [6]. BMP6 is one of the most potent regulators of osteoblast differentiation [19]. We showed here, BMP6 was highly expressed in BMMSCs from SS patients and disease mice, and enhanced osteogenic fate determination was observed in BMP6-treated BMMSCs. These findings suggested that high level of BMP6 in BMMSCs from SS patients and NOD mice may not contribute to the decreased osteogenic potential of BMMSCs.

A predominance of Th1 and Th17 cell responses and their products, notably INF-gamma and IL-17, in primary SS patients has been reported [20, 21]. MSCs can regulate the adaptive and innate immune systems by suppression of T cells and maturation of dendritic cells, reducing B-cell activation and proliferation, inhibiting proliferation and cytotoxicity of natural killer (NK) cells, and promoting the generation of regulatory T cells [22]. We found that BMMSCs significantly reduced the frequency of Th1 and Th17 cells in T cells-BMMSCs coculture system. BMP6 abolished this suppressive effect, demonstrating that BMMSCs suppressed Th1 and Th17 responses in SS.

Several soluble factors, including prostaglandin E2 (PGE2) [12], indoleamine-pyrrole 2,3-dioxygenase (IDO) [11], nitric oxide (NO) [23], and transforming growth factor-*β*1 (TGF-*β*1) [22], have been proposed to mediate this immunosuppressive effect. In the present study, we found that BMP6 can only downregulate the expression of PGE2 in BMMSCs. It has been known for over 30 years that PGE2 has a largely immunosuppressive role in T-cell activation and proliferation [24]. Here, we reported that BMP6 decreased PGE2 secretion in a T cells-BMMSCs coculture system and promoted T cell proliferation and Th1 and Th17 polarization, indicating that PGE2 is involved in the BMP6-induced impaired immunomodulatory ability of BMMSCs.

Id1 is a negative regulator of basic helix-loop-helix (bHLH) protein [25]. It was reported that Id1 promoter is a BMP-responsive element [26]. It is one of the most critical targets of BMPs and is responsible for various biological activities of BMPs [27–29]. Ids are also suggested to be involved in the determination of function of MSCs induced by BMPs [10]. We found that BMMSCs derived from the SS mice model expressed a higher level of BMP6 and Id1, and treatment with BMP6 upregulated Id1 gene expression in BMMSCs derived from control mice. Further experiments demonstrated that Id1 was involved in BMP6-induced impairment of BMMSC immunomodulatory function. Id1 was found to inhibit PGE2 secretion and stimulate T cell proliferation and Th1/Th17 differentiation in a T cells-BMMSCs coculture system. These data reveal a novel role of Id1 in BMP-mediated MSC function.

## 5. Conclusion

In conclusion, BMP6 was found to be expressed at a higher level in BMMSCs derived from SS patients as well as in animal models. BMP6 inhibited the immunomodulatory properties of BMMSCs by promoting T cell proliferation and Th1/Th17 differentiation. Mechanistically, BMP6 downregulated PGE2 and upregulated IFN-gamma via Id1. Neutralizing the BMP6 and knockdown Id1 could restore the BMMSC immunosuppressive function both *in vitro* and *in vivo*.

## Data Availability

The data used to support the findings of this study are available from the corresponding author upon request.

## Supplementary Materials

Supplementary Materials Supplementary Figure 1: Surface makers of BMMSCs.
