# Supplementary figures and images for: Bone Morphogenetic Protein 6 Inhibits the Immunomodulatory Property of BMMSCs via Id1 in Sjögren's Syndrome
Source: Stem Cells Int. 2018 Aug 2;2018:9837035. doi: 10.1155/2018/9837035 (PMC6098892; doi:10.1155/2018/9837035)

Supplementary Figure 1: Surface makers of BMMSCs

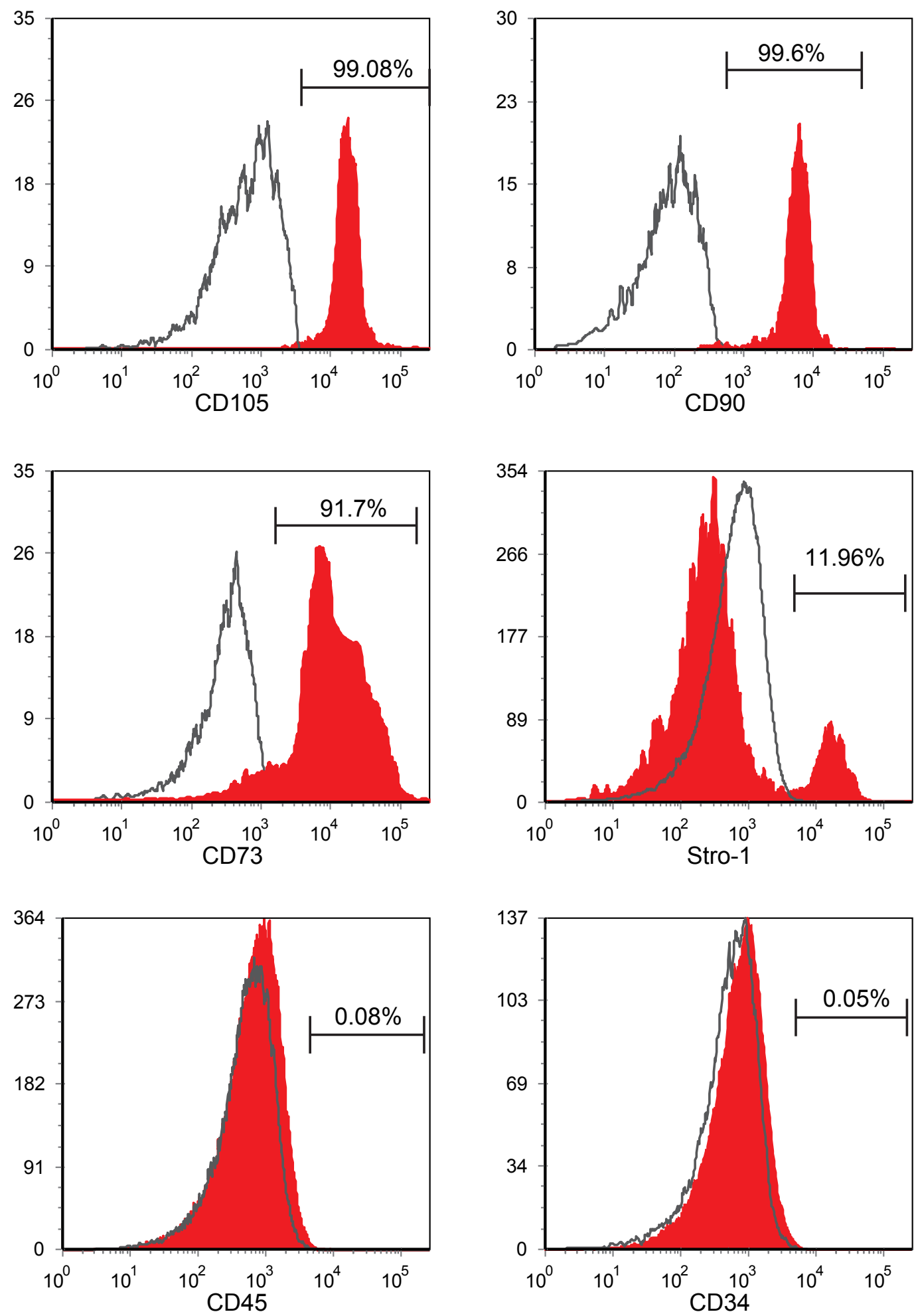

Supplement: Supplementary Materials — Supplementary Figure 1: Surface makers of BMMSCs. [file 9837035.f1.pdf]
